# Forced Convective Heat Transfer in Boundary Layer Flow of Sisko Fluid over a Nonlinear Stretching Sheet

**DOI:** 10.1371/journal.pone.0100056

**Published:** 2014-06-20

**Authors:** Asif Munir, Azeem Shahzad, Masood Khan

**Affiliations:** 1 Department of Mathematics, Quaid-i-Azam University, Islamabad, Pakistan; 2 Basic Science Department, UET, Taxila, Pakistan; Northwestern Polytechnical University, China

## Abstract

The major focus of this article is to analyze the forced convective heat transfer in a steady boundary layer flow of Sisko fluid over a nonlinear stretching sheet. Two cases are studied, namely (i) the sheet with variable temperature (PST case) and (ii) the sheet with variable heat flux (PHF case). The heat transfer aspects are investigated for both integer and non-integer values of the power-law index. The governing partial differential equations are reduced to a system of nonlinear ordinary differential equations using appropriate similarity variables and solved numerically. The numerical results are obtained by the shooting method using adaptive Runge Kutta method with Broyden’s method in the domain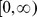. The numerical results for the temperature field are found to be strongly dependent upon the power-law index, stretching parameter, wall temperature parameter, material parameter of the Sisko fluid and Prandtl number. In addition, the local Nusselt number versus wall temperature parameter is also graphed and tabulated for different values of pertaining parameters. Further, numerical results are validated by comparison with exact solutions as well as previously published results in the literature.

## Introduction

The flow and heat transfer over stretching surfaces are relevant to many technological processes. The study of boundary layer flow and heat transfer induced by stretching heated surface has attracted considerable attention of many researchers. Some exemplary applications of such study are glass fibre production, the cooling and drying of paper while paper production, drawing of plastic films and so forth. Starting from the pioneering work of Sakiadis [1] numerous aspects of momentum and heat transfer over a stretching sheet have been considered. Some investigations dealing with the flow and heat transfer over nonlinear stretching surface are reported in Refs. [2]–[6].

All of the above mentioned studies are restricted to viscous fluids. However, there is a voluminous body of knowledge that testifies the wide occurrence of non-Newtonian fluids in industrial sector including food, pharmaceutical, polymer and plastic, mineral suspensions, cosmetics, personal care products, toiletries, construction materials and biological products [7]. In view of overwhelming practical utility of non-Newtonian fluids, several researchers have proposed numerous models for non-Newtonian fluids, including the Sisko fluid model [8]. The Sisko fluid is of much significance due to its adequate description of many non-Newtonian fluids over the most important range of shear rates. The three parametric Sisko model can be considered as a generalization of Newtonian and power-law fluids. The three constants in the model can be chosen with great ease for specific fluids and model is found to be good in predicting the shear thinning and shear thickening behaviors.

Extensive research has been commenced over past few years on the flow and heat transfer of non-Newtonian fluids over a stretching surface owing to its tremendous industrial utilization. Firstly, Schowalter [9] obtained the similar solutions for the boundary layer flow for power-law pseudoplastic fluids. Jadhav and Waghmode [10] analyzed the heat transfer to power-law fluid over a permeable flat plate with heat flux boundary condition. Howel *et al.* [11] considered the laminar flow and heat transfer of a power-law fluid over a stretching sheet. Hassanien *et al.* [12] numerically analyzed the flow and heat transfer for a stretching sheet for non-uniform temperature distribution. Able *et al*. [13] studied the flow and heat transfer to a power-law fluid over a stretching sheet by considering variable thermal conductivity and heat source.

Polymeric suspensions such as waterborne coatings are identified to be non-Newtonian in nature and are proven to follow the Sisko fluid model [14]. The Sisko fluid model was originally proposed for high shear rate measurements on lubricating greases [15]. Khan *et al*. [16] examined the steady flow and heat transfer of a Sisko fluid in annular pipe. Then, Khan and Shahzad [17], [18] developed the boundary layer equations for Sisko fluid over planer and radially stretching sheets and found the analytical solutions for only integral values of the power-law index. The utmost studies relating to the heat transfer of Sisko fluid involve only one dimensional flows and literature survey indicates that no work has so far been communicated with regards to heat transfer in a boundary layer flow for Sisko fluid over a nonlinear stretching sheet with variable surface temperature and variable heat flux.

The prescribed surface temperature and prescribed surface heat flux are the generalizations of constant surface and constant heat flux thermal boundary conditions. Such boundary conditions are encountered in polymer processing industry, where the surface temperature may be an arbitrary function of time or space. The prescribed heat flux may be taken when there is surface heat generation via solid-solid friction, as in frictional welding and conveying of solids in screw extruders, is an example. Moreover, certain types of intensive radiation or convective heating that are weak functions of surface temperature can also be treated as a prescribed surface heat-flux boundary condition [19].

This paper brings to focus the heat transfer in a boundary layer flow of Sisko fluid over a nonlinear stretching sheet with variable wall temperature and heat flux boundary conditions. Khan and Shahzad [18] in their work only considered the integral values of the power-law index. In this investigation, the non-integer values are also taken into consideration.

## Formulation of the Problem

### Flow Analysis

We consider the steady, laminar and incompressible flow of a Sisko fluid impinging normal to a stretching sheet coinciding with the plane 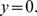 The flow is induced due to stretching of the sheet along the *x*–direction while keeping the origin fixed with velocity 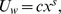 where 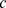
and *s* are positive real numbers relating to stretching of sheet. For a two-dimensional flow, we assume the velocity field of the form
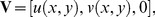
(1)


Where 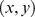 denotes the Cartesian coordinates along and perpendicular to the sheet, 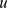 and 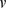 are the velocity components of fluid along the 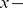
and 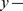
directions, respectively.

Using the simplifying assumptions, the momentum equation characterizing the steady boundary layer flow takes the form 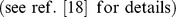


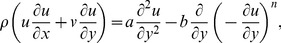
(2)


Where *a, b* and 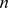 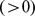 are the material constants.

The flow is subject to the boundary conditions
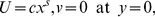
(3)

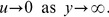
(4)


In view of the similarity transformations [18]

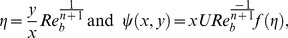
(5)and after mathematical simplification, we obtain the following problem
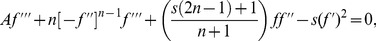
(6)

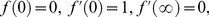
(7)where prime denotes the differentiation with respect to 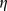
and the dimensionless quantities are defined by
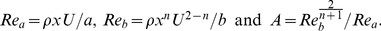
(8)The physical quantity of major interest is the local skin friction coefficient and in dimensionless form is given by 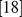


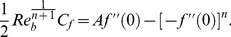
(9)


### Heat Transfer Analysis

By using the usual thermal boundary layer approximations, neglecting viscous dissipation and heat generation, the energy equation for temperature field *T = T(x,y)* is given by
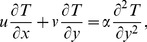
(10)where 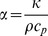 is the thermal diffusivity with 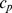 as the specific heat of fluid at constant pressure and 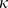 the thermal conductivity.

Two kinds of thermal boundary conditions are considered in heat transfer analysis, and they are treated separately in the following sections.

### Prescribed Surface Temperature (PST Case)

In this case, the thermal boundary conditions are
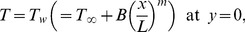
(11)

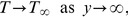
(12)where 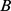 is a constant, 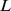 the characteristic length, 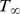
the constant fluid temperature far away from the sheet, 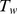 the wall temperature and 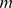 the wall temperature parameter.

Defining the non-dimensional temperature 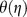 by
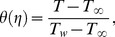
(13)and using Eqs. 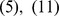 and 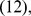 Eq. 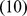 takes the form
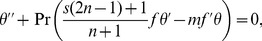
(14)where 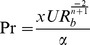 is the generalized Prandtl number.

The boundary conditions for 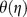 follow from Eqs. 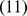 and 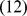 are
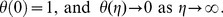
(15)


The rate of heat transfer at the sheet surface is
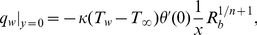
(16)which on simplification reduces to
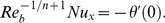
(17)where 
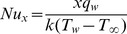
 is the local Nusselt number.

### Prescribed Heat Flux (PHF Case)

In PHF case, the dimensionless temperature 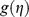 is defined as
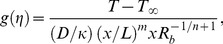
(18)with the following boundary conditions
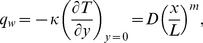
(19)

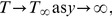
(20)where 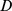 is a constant, and 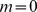 leads to a constant heat flux case.

Using Eqs.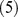 and 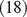 in Eq. 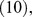
one finds that
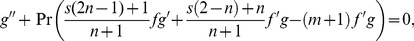
(21)




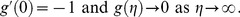
(22)


The local heat transfer coefficient is
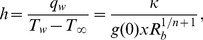
(23)resulting in the local Nusselt number given by
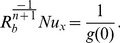
(24)with 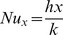 as the local Nusselt Number.

### Exact Solutions for Particular Cases

Since Eq. 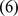 has simple exact solution to a special case, namely 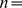 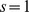 (see ref. 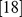 for detail). For this case with 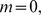 Eq.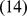 reduces to
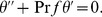
(25)


The exact solution of Eq. 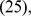 in terms of the incomplete Gamma function, satisfying boundary conditions 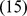 is
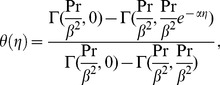
(26)where 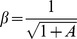 and 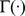 the incomplete Gamma function.

For 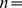 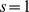 and 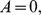
Eq. 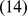 reduces to
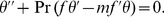
(27)


The exact solution of the above equation satisfying the boundary conditions 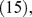 in terms of Kummer’s function, is expressed as [20]

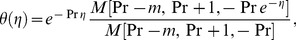
(28)


Where 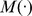 is the Kummer’s function.

When 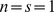 and 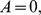 Eq.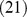 has exact solution satisfying boundary conditions 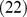 of form [20]

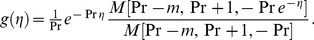
(29)


### Solution Methodology

In general it is very difficult to find the exact analytical solution of non-linear two point boundary value problem (6), (14) and (21) along with boundary conditions (7), (15) and (22). Therefore, these problems are solved numerically by the shooting technique. The equations are firstly written as a system of five first order ordinary differential equations. Then the corresponding initial value problems are solved by the adaptive Runge-Kutta method. The initially guessed values 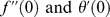 are refined iteratively using the Boryden’s method to satisfy boundary condition at infinity. The iterative process is terminated when the absolute error is less than the tolerance 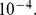


## Numerical Results and Discussion

**Figure 1:**
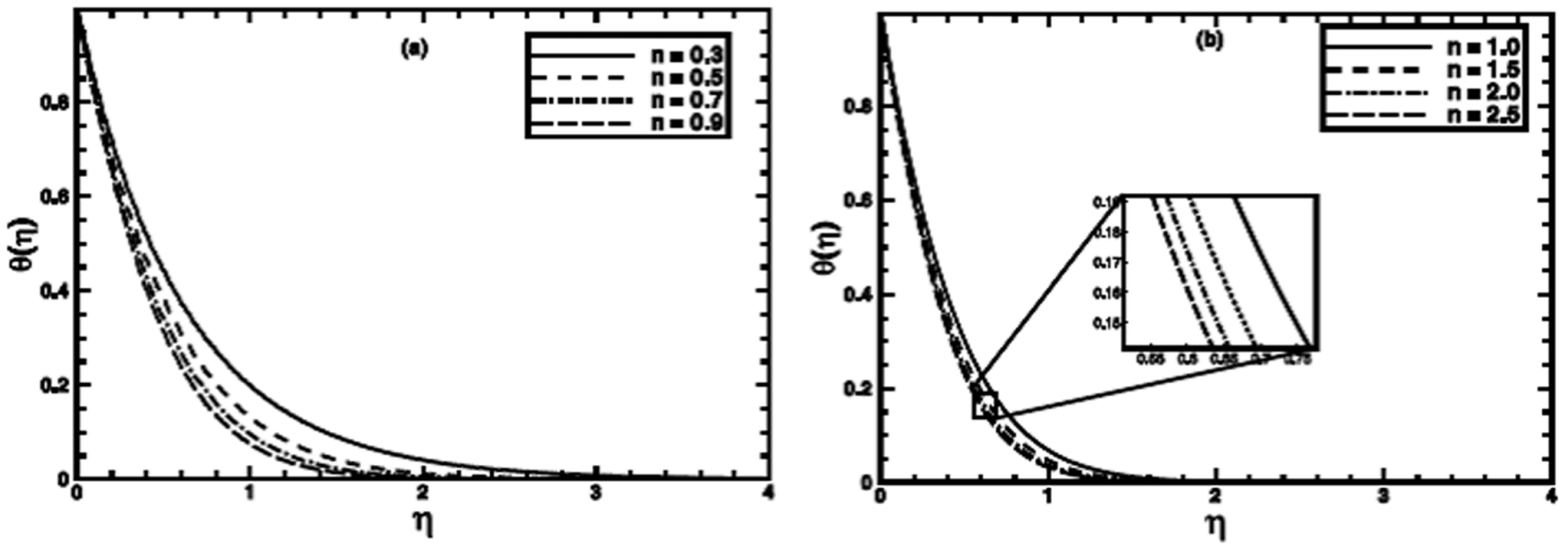
The temperature profile 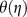 in the PST case for different values of the power-law index 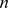 when 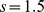, 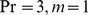 and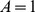 are fixed.

**Figure 2:**
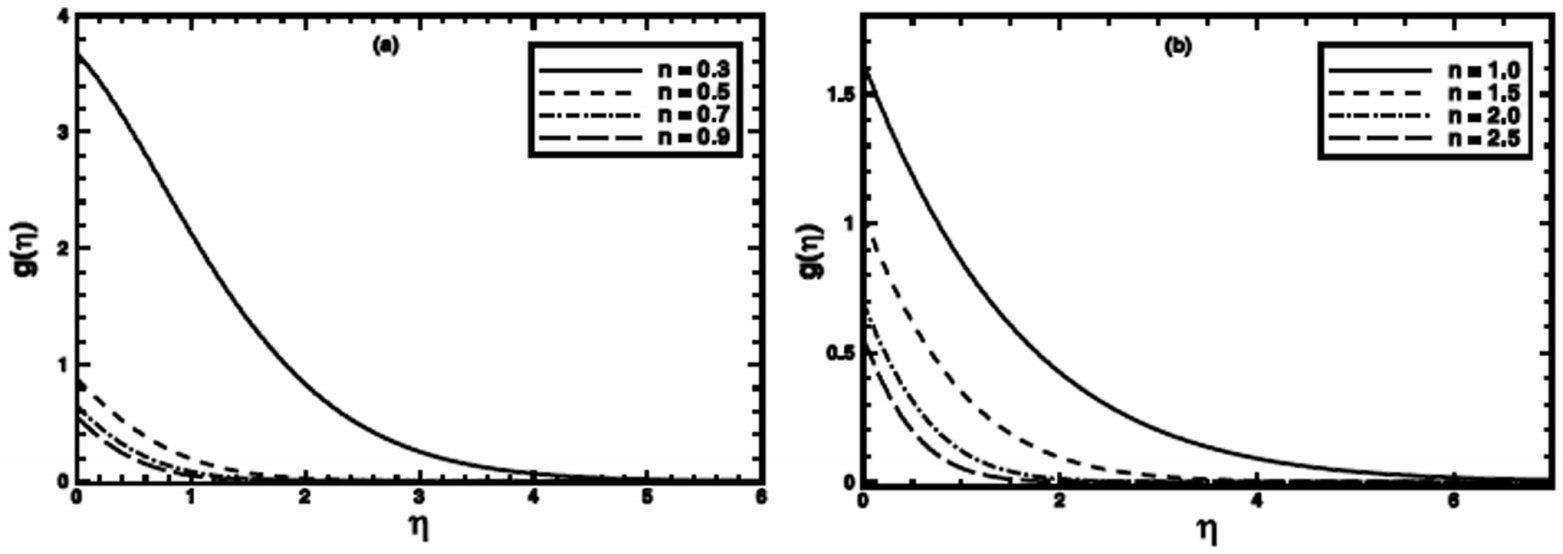
The temperature profile 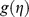 in the PHF case for different values of the power-law index 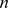 when 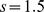, 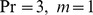 and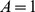 are fixed.

**Figure 3:**
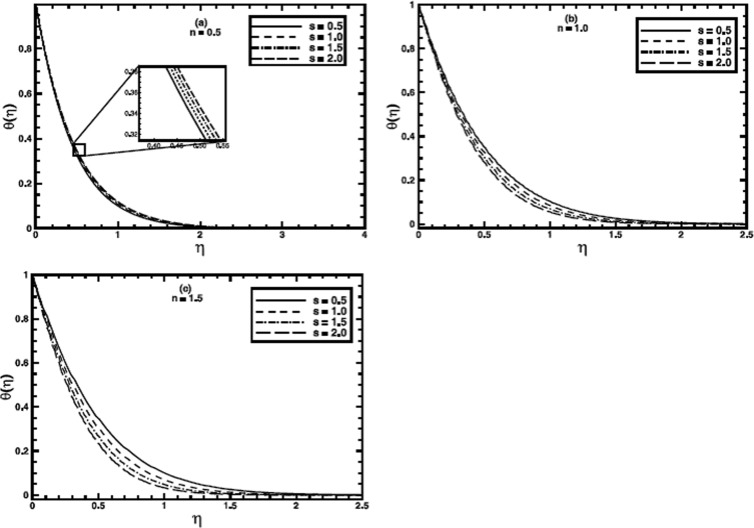
The temperature profile 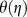 in the PST case for different values of the stretching parameter *s* when 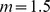, 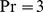 and 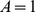 are fixed.

**Figure 4:**
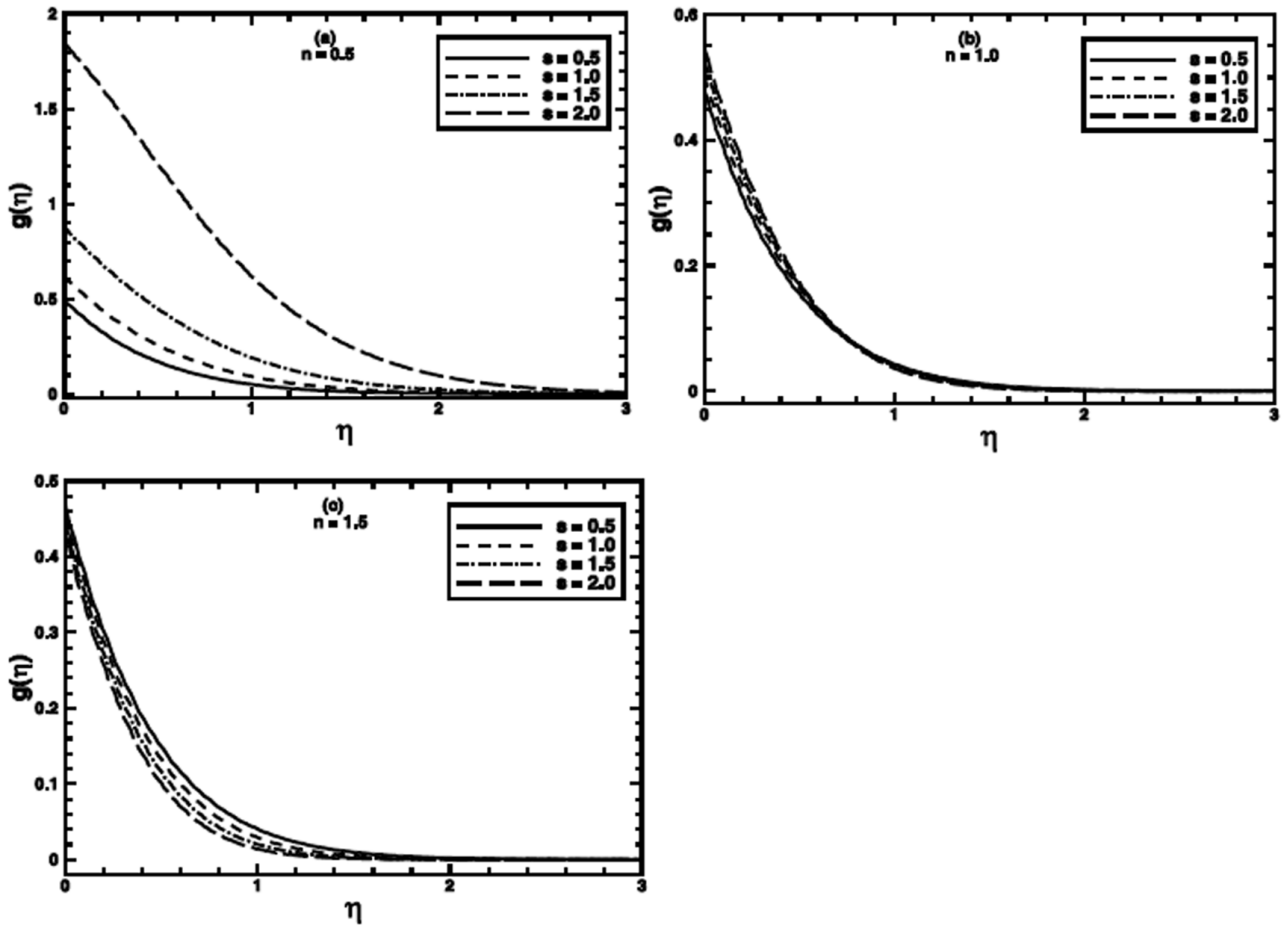
The temperature profile 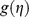 in the PHF case for different values of the stretching parameter *s* when 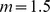, 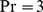 and 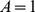 are fixed.

**Figure 5:**
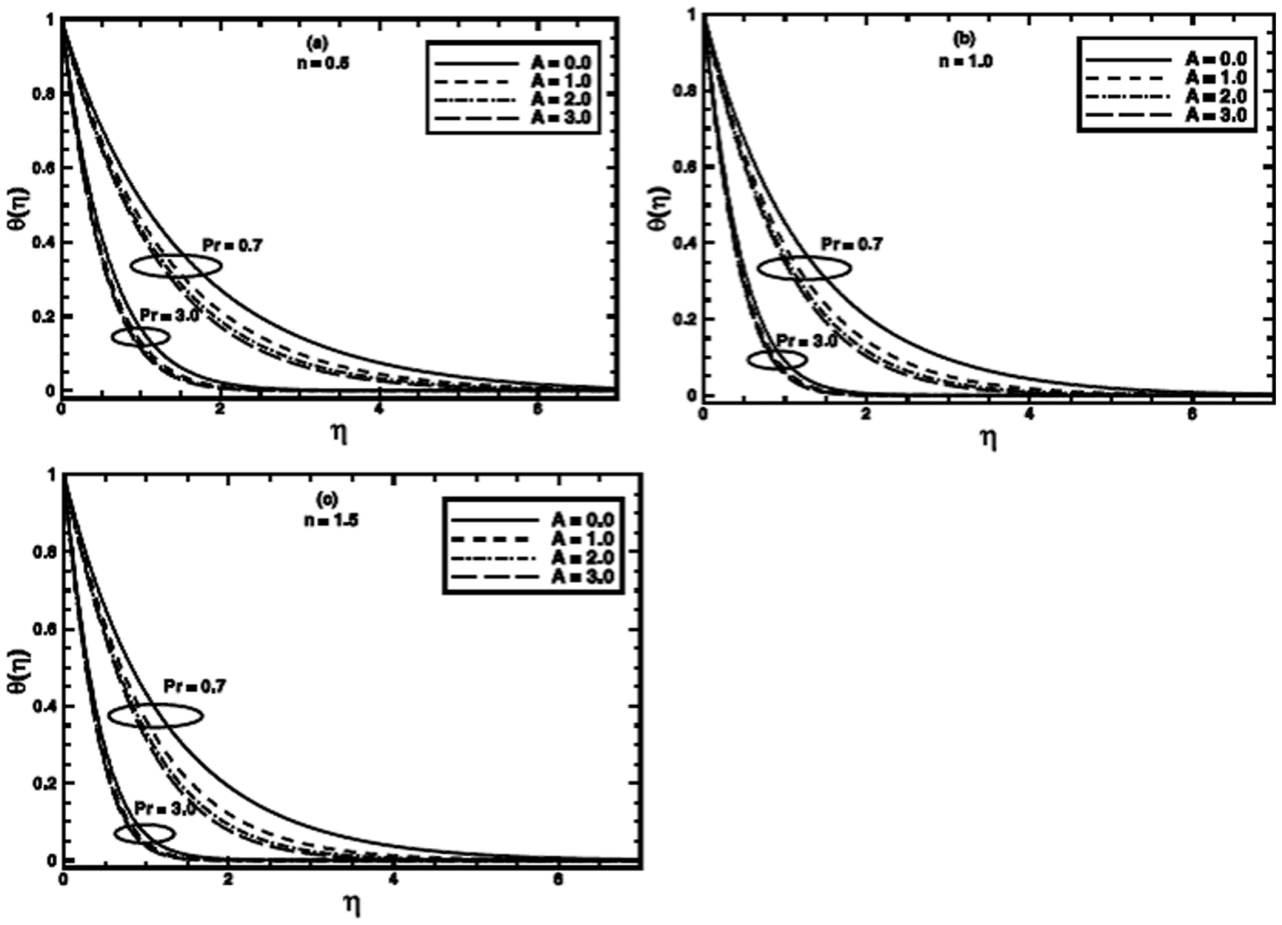
The temperature profile 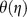 in the PST case for different values of the material parameter *A* when 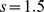 and 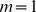 are fixed.

**Figure 6:**
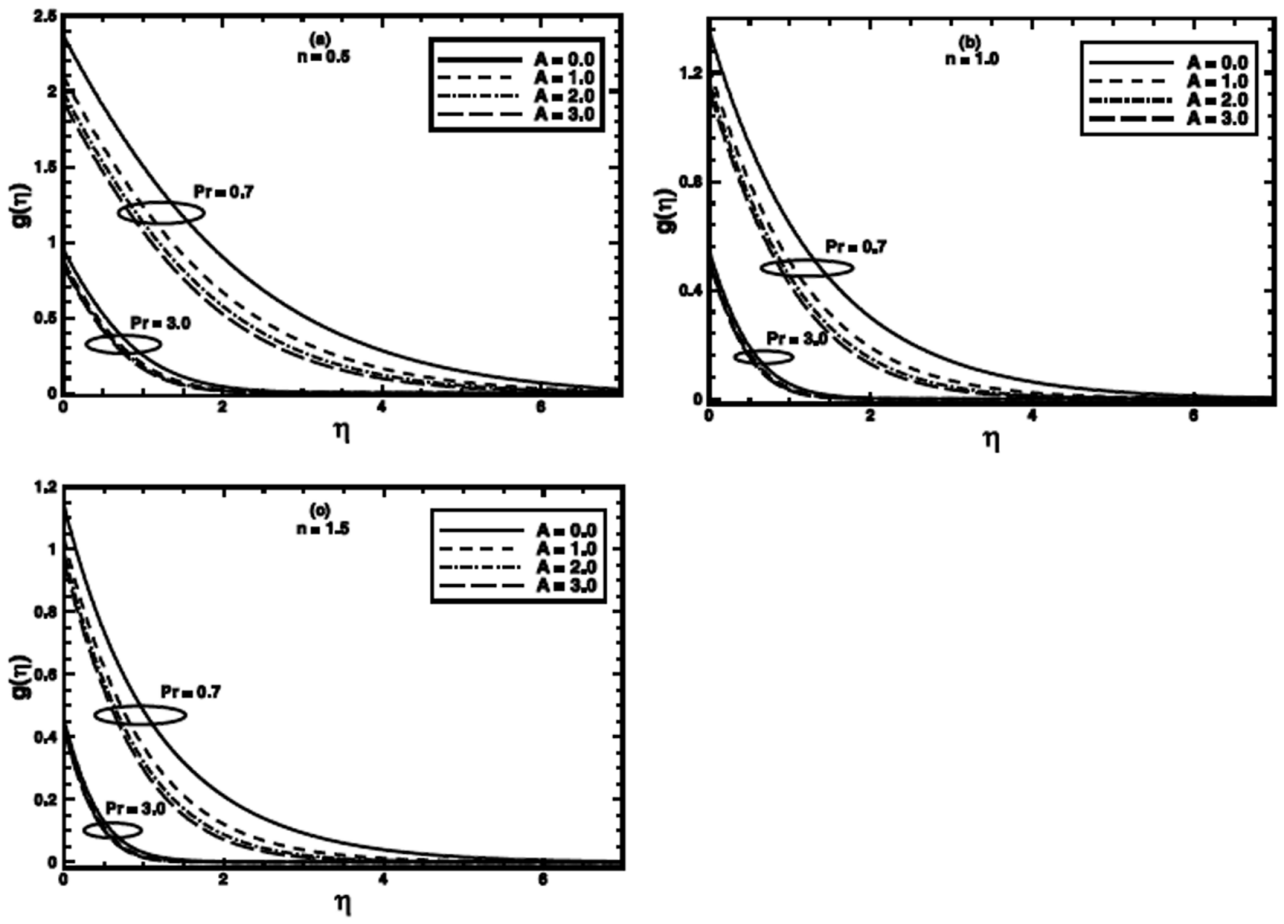
The temperature profile 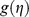 in the PHF case for different values of the material parameter *A* when 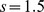 and 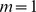 are fixed.

**Figure 7:**
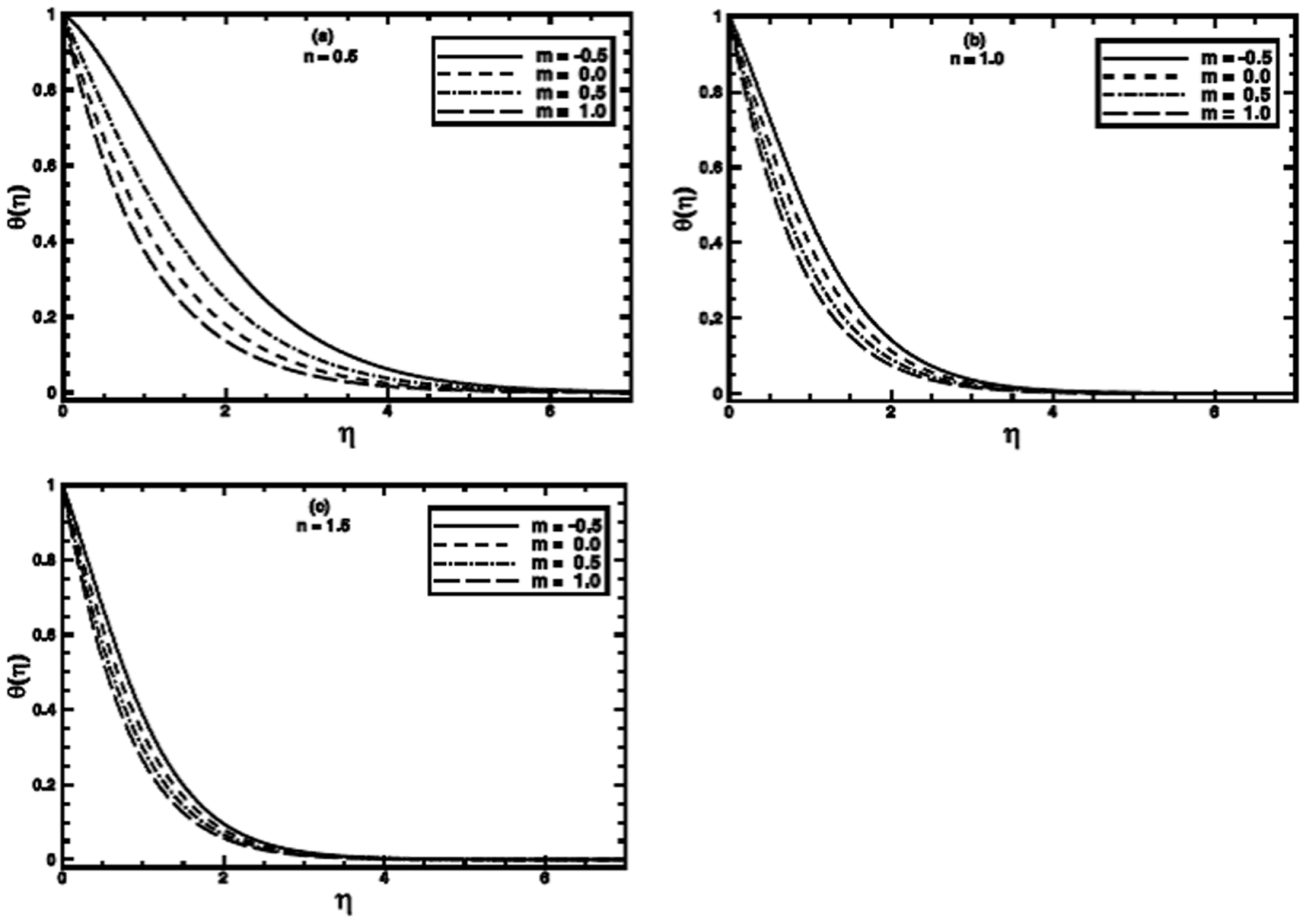
The temperature profile 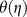 in the PST case for different values of the temperature parameter *m* when 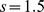, 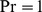 and 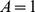 are fixed.

**Figure 8:**
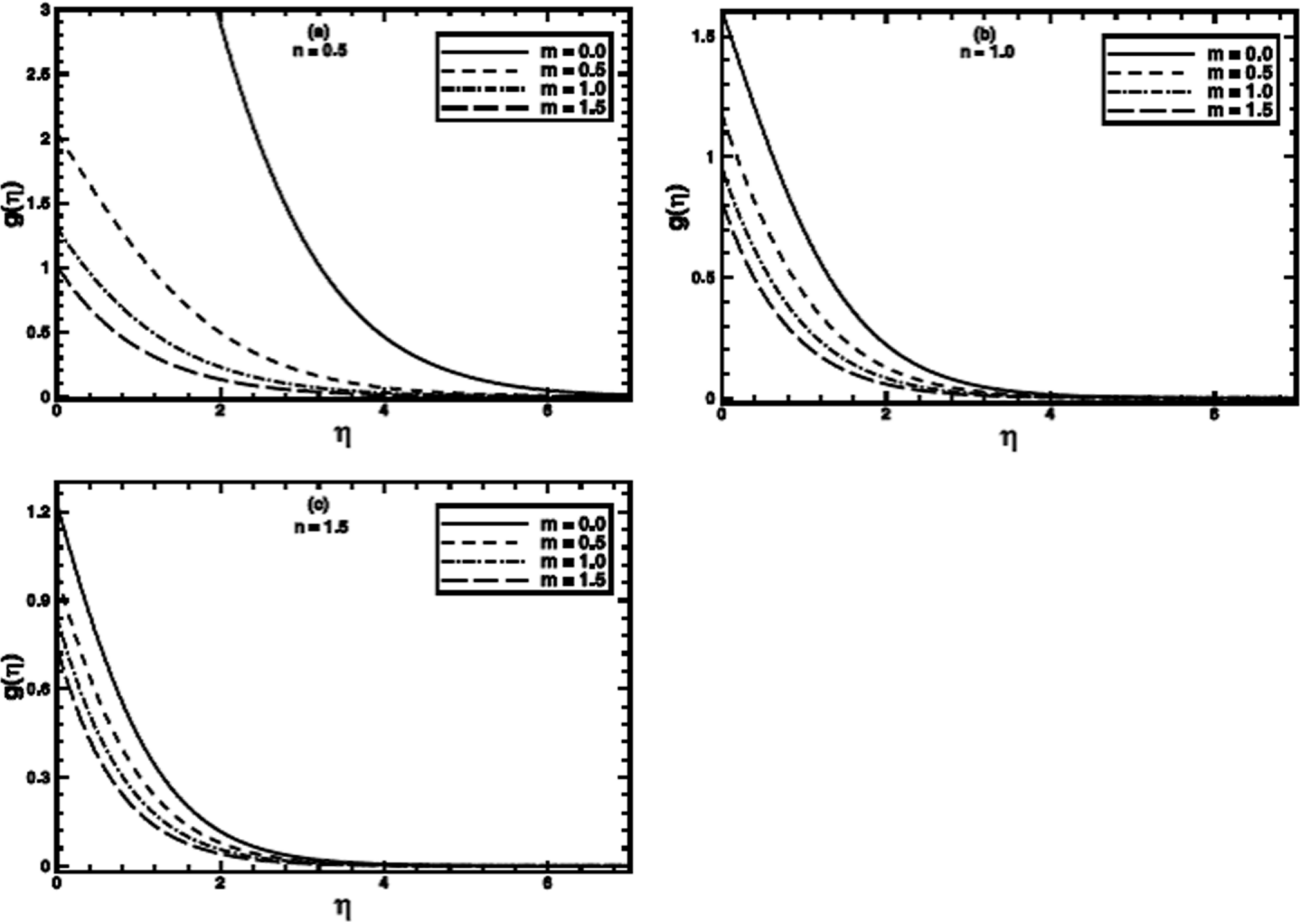
The temperature profile 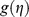 in the PHF case for different values of the temperature parameter *m* when 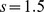, 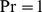 and 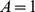 are fixed.

**Figure 9:**
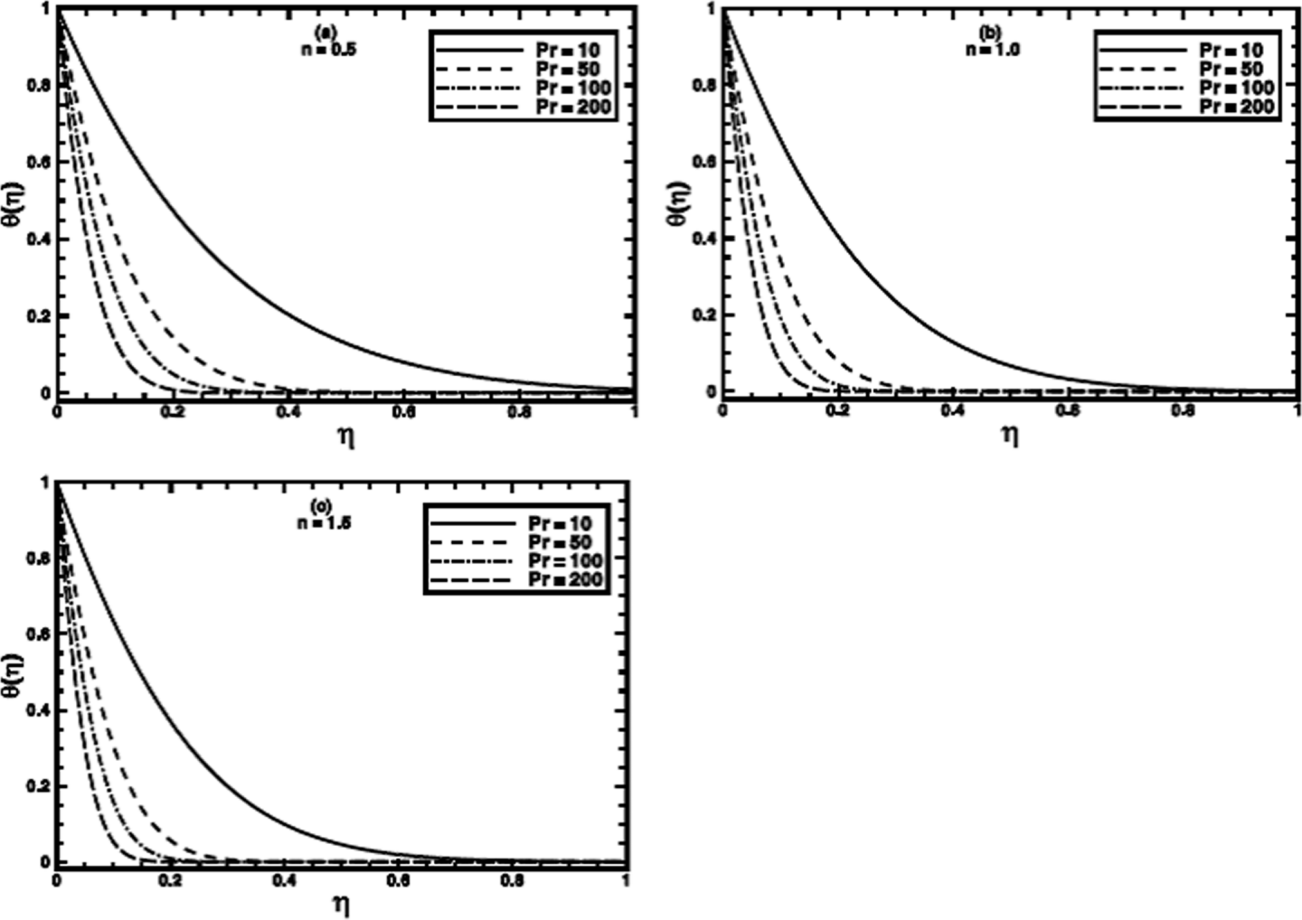
The temperature profile 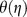 in the PST case for different values of the Prandtl number Pr when 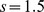, 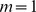 and 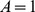 are fixed.

**Figure 10:**
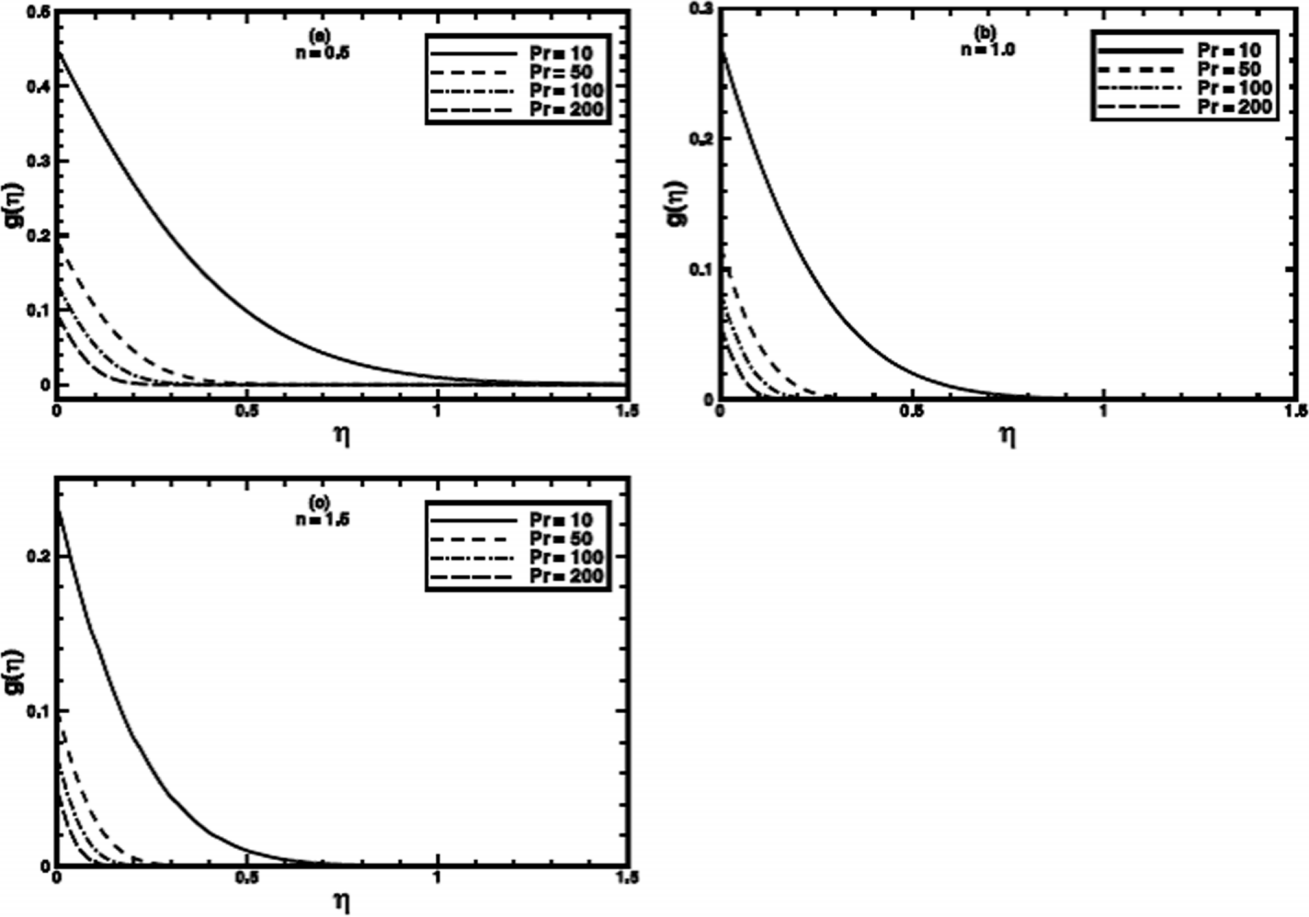
The temperature profile 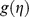 in the PHF case for different values of the Prandtl number Pr when 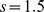, 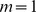 and 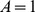 are fixed.

The main aim of the present study is to investigate the heat transfer to a Sisko fluid over a nonlinear stretching sheet with the non-isothermal wall temperature and variable heat flux boundary conditions. In order to comprehend the heat transfer phenomena in detail, Eqs. 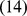 and 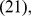 with their respective boundary conditions, are solved numerically and the results are displayed through graphs. Mainly, the effects of the power-law index 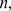 stretching parameter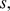 Prandtl number 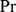, material parameter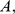 and surface temperature parameter 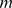 are investigated in detail both for PST and PHF cases. Moreover, the heat transfer aspects are explored in terms of the local Nusselt number at the wall. Figures 1–10 give the various perspectives of heat transfer for both PST and PHF cases. The values of the local Nusselt number are recorded in tables 1 and 2 for both PST and PHF cases, respectively.

**Table 1:** The numerical values of the Local Nusselt number in the PST case when 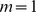 and 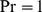 are fixed.

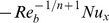				
*A*	*s*	*n* = 0.5	*n* = 1.0	*n* = 1.5
1	1	1.01162	1.07693	1.10805
2	1	1.04162	1.11064	1.14661
3	1	1.05911	1.12991	1.16897
1	2	0.96658	1.14410	1.23540
2	2	1.00436	1.18608	1.28120
3	2	1.02653	1.21105	1.30854

**Table 2:** The numerical values of the Local Nusselt number in the PHF case when 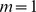 and 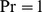 are fixed.

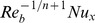				
*A*	*s*	*n* = 0.5	*n* = 1.0	*n* = 1.5
1	1	0.86916	1.07706	1.17826
2	1	0.89766	1.11033	1.21713
3	1	0.91446	1.13041	1.23968
1	2	0.27308	0.97012	1.23540
2	2	0.29081	1.00985	1.28121
3	2	0.30209	1.03362	1.30854

The effect of the power-law index 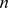 on temperature profile 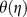 is presented in figures 1 and 2 for both PST and PHF cases, respectively. A wider range of the power law index *n* is spanned in the analysis 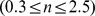 covering from highly shear thinning to thickening fluids.

Most polymeric melts and solutions have value of the power law index in the range 0.3–0.7 [21], moreover, typical starch-in-water and corn flour-in-water dispersion have values of power-law index, 1.4–1.6 [22]. The figure 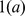 shows that the temperature profile increases when the power-law index is decremented below one. This increase can be attributed to the fact that the reduction in viscosity favors the heat transfer. The increase is large for smaller values of 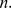 The temperature profile also decreases as the value of 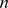 is incremented gradually (figure 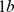) for 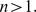 A comparison of the two patterns reveals a strong dependence of heat transfer on flow behavior index for 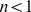
and weaker when 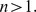 The figures 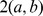 elucidate the temperature profiles for the PHF case for 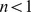 and 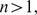 respectively. It is quite clear from these figures that the temperature profiles decrease when the value of the power-law index is increased for shear thinning 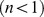 and shear thickening 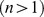 regimes. These figures show that, in the PHF case, the behavior of temperature profile with change in 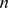 is similar to the PST case. However, a stronger dependence of the temperature profile can be noticed in the PHF case. Both the figures put in an evidence that an augmentation in the value of the power-law results in a decrease of the thermal boundary layer thickness.

The stretching parameter 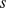 affects the temperature profile and thermal boundary layer by virtue of imparting shear stress at the boundary. A broader range 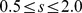 is included in the analysis, including 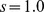 for linear stretching. Its effects on heat transfer for both boundary conditions are presented in figures 3 and 4 respectively. For the PST case, when the value of the power-law index (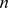 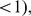 the effect of the stretching parameter 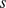 on heat transfer to the fluid is very meager; however, the thermal boundary layer thickness increases a bit as the value of 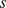 is incremented progressively. We can ascribe this increase to the amplification of shear stress with each increment in *s*. For shear thinning regime 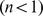 the enhancement of shear stress lowers the effective viscosity, that favors the heat transfer. Although, for 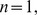 the effect of 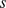 on heat transfer is noticeable 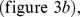 but a contrary behavior for the temperature and thermal boundary layer thickness is observed. Figure 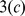 presents the effect of the stretching parameter 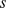 on the temperature profile for 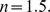 The temperature and thermal boundary layer decrease and 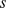 affects the thermal boundary layer thickness substantially for this case. Figure 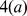 depicts that the temperature profile raises as the value of 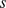 is augmented for shear thinning (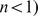 regime. The temperature near the wall grows quite prominently as the value of 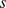 is incremented. The character of temperature profile, when 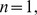
and stretching parameter 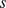 varied is shown in figure 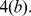 This figure reveals that on increasing the value of the stretching parameter 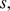 the temperature profile increases near the wall and then decreases away from the wall. For shear thickening 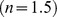 regime
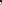
the temperature profile and corresponding thermal boundary layer show a diminution as the value of 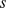 is raised (figure 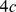).

The effect of the material parameter 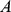 on the temperature profile for nonlinear stretching is presented in figures 5 and 6 respectively, for PST and PHF cases. These figures also make a comparison amongst the temperature profiles of the Newtonian fluid 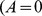 and 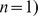 and the power-law fluid 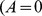 and 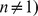 with those of the Sisko fluid 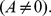 Figures 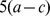 reveal that the temperature profile and corresponding thermal boundary layer thickness depress in each case with increasing value of the material parameter 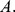 It can be noticed from the sketches that the effect of 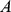 on the thermal boundary layer is pronounced for low Prandtl number as compare to higher one. Figures 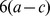 demonstrate that the temperature profile and corresponding thermal boundary layer thickness also decrease with an increase in the value of 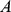 for different values of power-law index 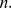 Effects are more prominent for lower Prandtl numbers.

The influence of the wall temperature parameter 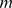 for both PST and PHF cases on the temperature distribution and thermal boundary layer thickness is presented in figures 7 and 8 respectively. Numerical solutions are sought in the range 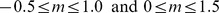 for PST and PHF cases, respectively. These ranges are selected on the basis of physically acceptable solutions. From these figures, it is observed that as the value of 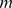 is incremented progressively from negative to positive, the temperature and thermal boundary layer thickness decrease as shown for some values of the power-law index 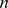 But, this effect turn out to be diminish for larger values of *n*.

The Prandtl number 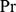 of a fluid plays a dominant role in forced convective heat transfer. The computations are carried out for large Prandtl number, many non-Newtonian fluids exhibit a value of Prandtl number as high as 100 or even greater [23], [24]. Its effect for the PST case for different values of power-law index 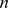 is shown in figures 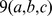. It is notified that the heat transfer process is augmented prominently by thinning the thermal boundary layer thickness when
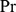
is increased. The augmentation can be ascribed to the enhanced momentum diffusivity for larger Prandtl number and the heat transfer mainly occurs due to advection. The same qualitative aspects are observed for the PHF case as shown in figures 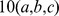.

**Figure 11:**
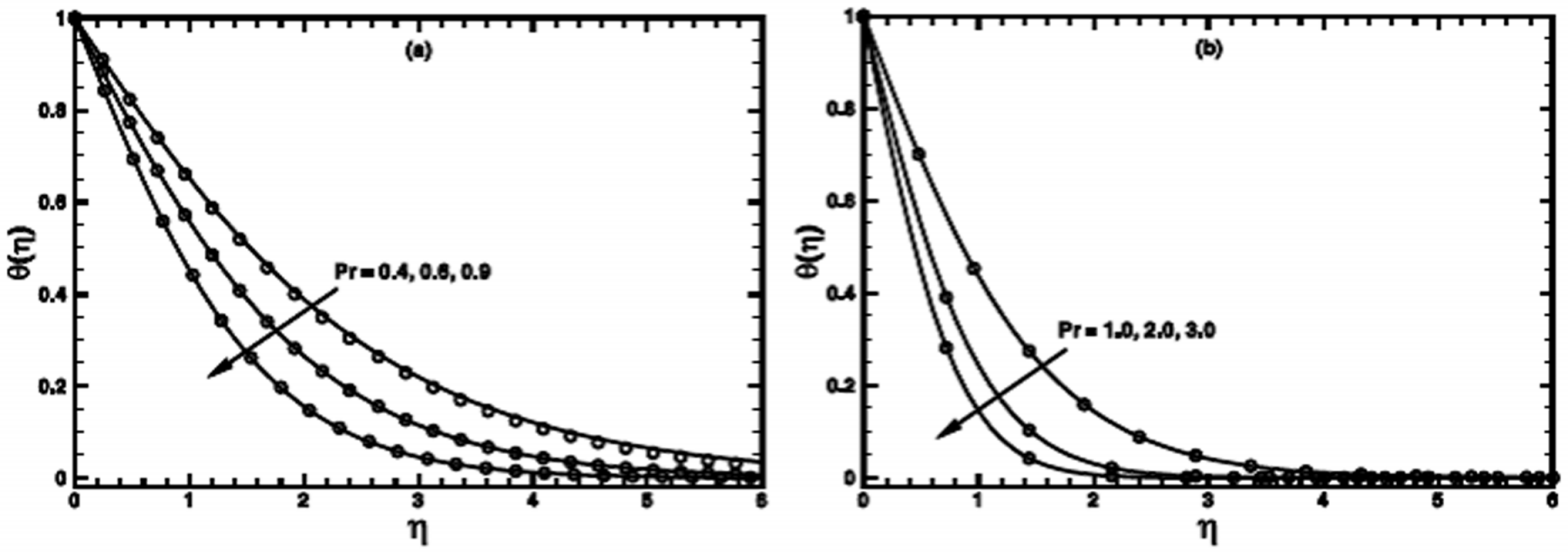
A comparison of the exact and numerical results (solid line numerical results and open circles exact results) in the PST case when 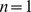, 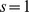 and 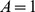 are fixed.

**Figure 12:**
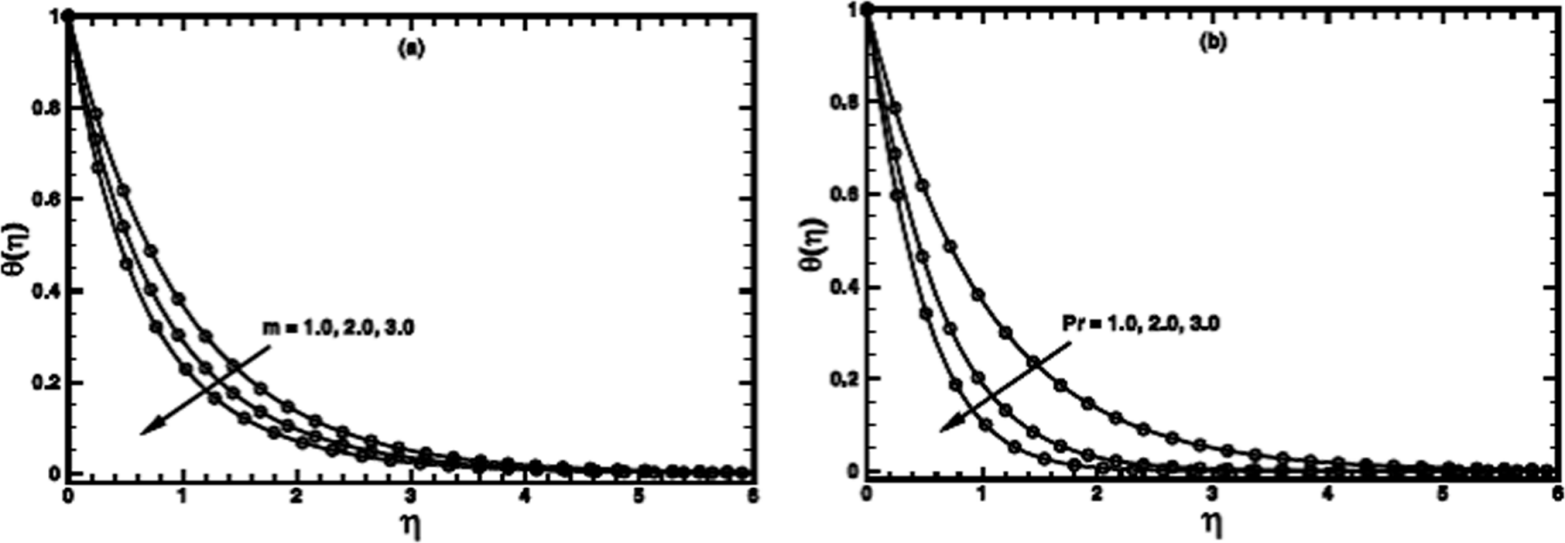
A comparison of the exact and numerical results (solid line numerical results and open circles exact results) in the PST when 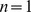, 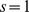
and 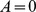 are fixed.

**Figure 13:**
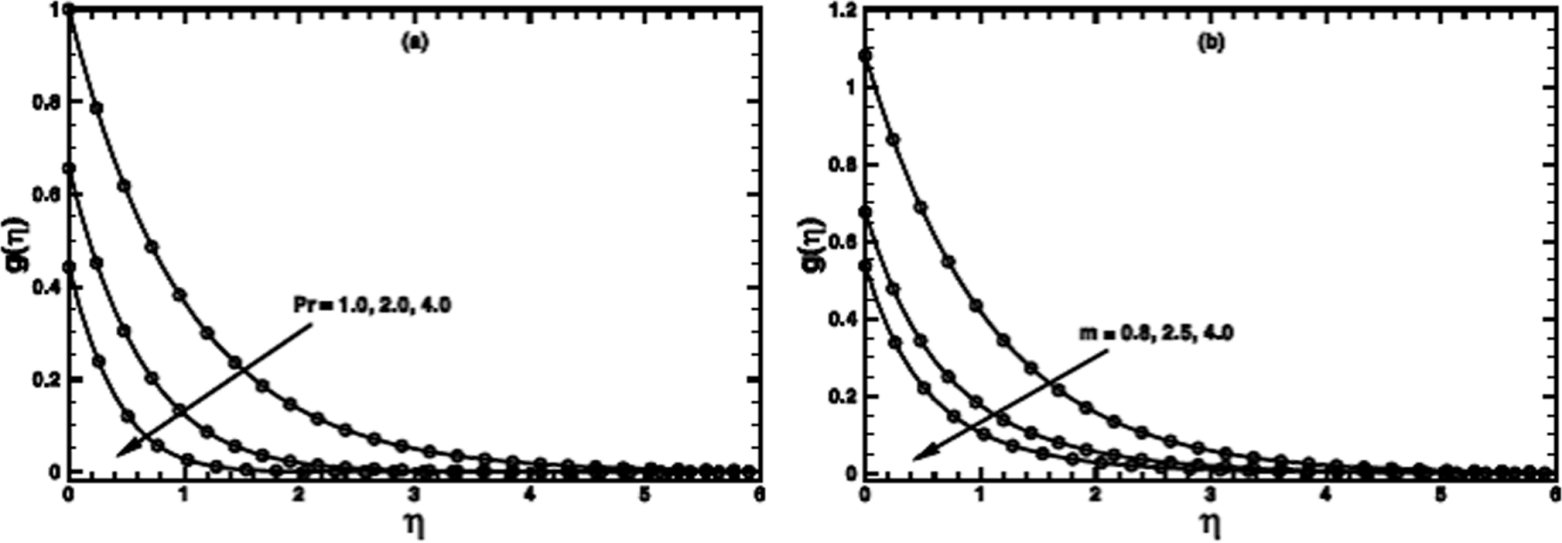
A comparison of the exact and numerical results (solid line numerical results and open circles exact results) in PHF case when 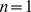, 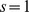 and 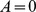 are fixed.

The numerical results are also compared with those of exact ones as special cases of the problem (figures 11–13). An excellent agreement confirms the credibility of our numerical solutions.

**Figure 14:**
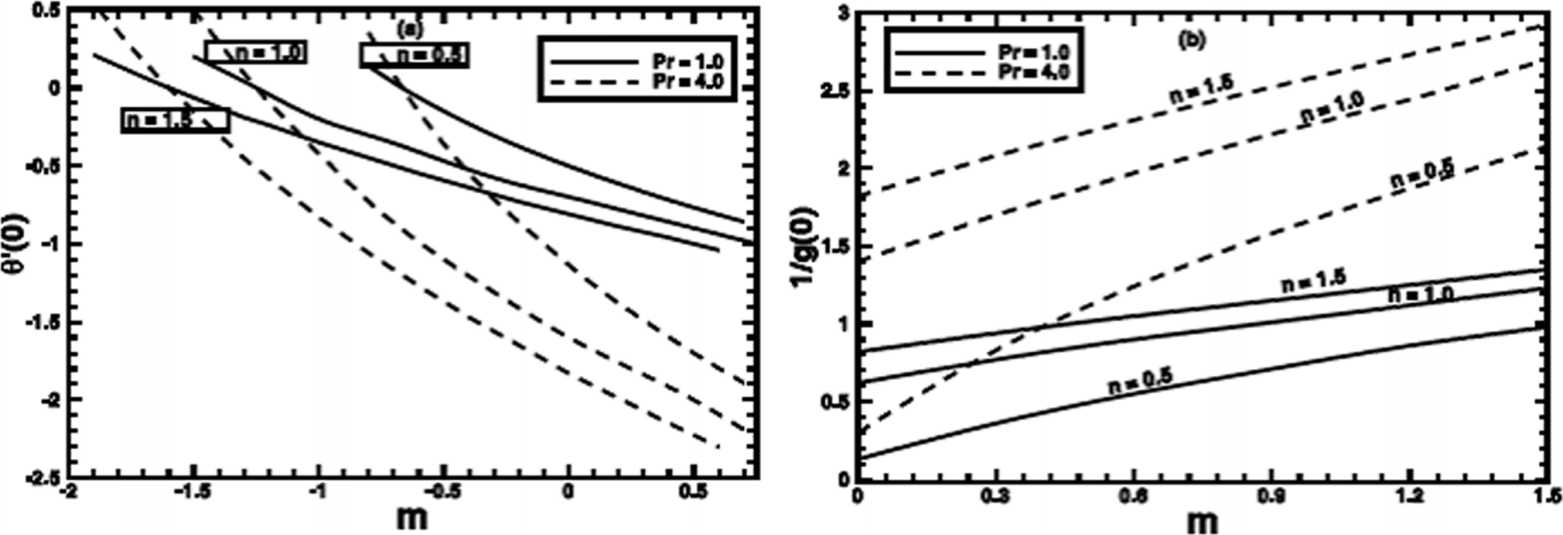
The variation of the local Nusselt number with *m* for some value of the power-law index *n* and Prandtl number Pr.

Figure 14(*a*) depicts the variation of the local Nusselt number 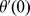 with change in wall temperature parameter 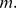 It is clearly observed from the plots that the heat transfer at the wall increases rapidly for the fluids with greater Pr. Moreover, the fluids with 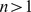
carry larger heat out of heated surface. These plots also give the value of 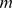
where the value of 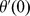 approaches to zero. This value of 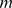 is strong function of the power-law index 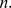 Figure 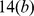 illustrates the effect of 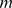 on the local Nusselt number for the Sisko fluid (both for shear thinning and shear thickening regimes). The Prandtl number affects the heat transfer in same fashion as previous one, but here more heat is transferred for each value of 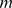 as compared to the PST case.

Table 1 briefly shows the trend in the local Nusselt number for shear thinning and thickening fluids when the material parameter 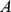 and stretching parameter 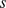 are varied for the PST case. It can be noticed from this table that the local Nusselt number shows a boost with an each increment of 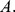 It is also clear that there is much boost in the local Nusselt number for flow behavior index 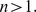 Table 2 displays the same informations and qualitatively similar trends are observed for the PHF case. Also, in table 3, a comparison with the obtained results of Chen [25] is made and excellent agreement is found.

**Table 3:** A comparison of the local Nusselt number in the PHF case.

	Chen [25]		Present study	
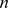	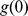	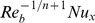	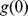	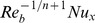
0.5	0.659092	1.517239	0.659091	1.517019
1.0	0.590316	1.694009	0.590312	1.694001
1.5	0.561785	1.780041	0.561781	1.780040

## Conclusions

The analysis in this article was focused on heat transfer in a Sisko fluid over a nonlinear stretching sheet both for variable temperature and heat flux boundary conditions. Numerical solutions are obtained and verified as special cases of the problem with exact results. Our computations have indicated that:

- A rapid decrease in the thermal boundary layer thickness was observed for Sisko fluid with shear thinning properties with PHF boundary condition.
- Thermal boundary layer thickness was increased for power-law-index 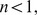 and decreased for 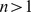 when stretching parameter 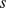
was increased. The effects were more noticeable for PHF case.
- Effects of the material parameter of the Sisko fluid were more prominent at lower values of Prandtl number.
- The thermal boundary layer thickness was decreased, when the wall temperature parameter was increased. This decrease was more rapid for PHF case with power law index 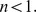

- Thinning of the thermal boundary layer was observed for increasing Pr. Such effects were dominant for power-law index 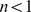 with PHF boundary condition.
- The local Nusselt number was increased for incremented values of the wall transfer parameter 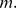
